# The Effect of Physical Exercise on Non-Oncological Musculoskeletal Chronic Pain and Its Associated Biomarkers: Systematic Review on Randomized Controlled Trials

**DOI:** 10.3390/life15091413

**Published:** 2025-09-08

**Authors:** Israel Castillo-Bellot, Ana María Peiró, Thomas Zandonai

**Affiliations:** 1 Department of Pharmacology, Pediatrics, and Organic Chemistry, Miguel Hernández University of Elche, 03550 Alicante, Spain; isra.castillo04@gmail.com (I.C.-B.); apeiro@umh.es (A.M.P.); 2 Addiction Science Laboratory, Department of Psychology and Cognitive Science, University of Trento, 38068 Rovereto, Italy

**Keywords:** chronic pain, musculoskeletal disorders, physical exercise, biomarkers

## Abstract

**Objective:** Non-oncological musculoskeletal chronic pain has a high prevalence and is a cause of disability, reduced quality of life, and significant economic impact. Physical exercise is presented as a treatment option; however, pain measurement remains a challenge, and various biomarkers are potential candidates to objectify this process. This systematic review aims to study the effect of physical exercise on non-oncological musculoskeletal chronic pain and its associated biomarkers based on randomized controlled trials. **Methods:** A search for randomized controlled trials was conducted in the PubMed, Web of Science, and Scopus databases based on the established inclusion and exclusion criteria, along with a risk of bias assessment following the recommendations of the Cochrane Collaboration. **Results:** Five studies investigated various physical exercise interventions and their effects on biomarkers linked to chronic pain. Exercise consistently reduced self-reported pain, though no clear overall correlation with biomarker changes was found. However, significant associations emerged for specific biomarkers, particularly inflammatory markers and those identified through structural and functional brain imaging, suggesting potential mechanisms underlying pain modulation. **Conclusions:** The findings suggest that identifying chronic pain variations through biomarkers requires selecting markers linked to immune activity or brain processes. More randomized controlled trials with sufficient sample sizes and rigorous methodologies are needed. Despite this, physical exercise remains a valuable intervention for managing non-oncological musculoskeletal chronic pain. Additionally, it holds potential as a tool for uncovering novel biomarkers that may contribute to the objectification and understanding of chronic pain mechanisms.

## 1. Introduction

Chronic pain is defined by the International Association for the Study of Pain (IASP) as “an unpleasant sensory and emotional experience associated with, or resembling that associated with, actual or potential tissue damage” [1], and clinically, it is defined as pain lasting more than 3 months [2]. Unlike acute pain, chronic pain offers little evolutionary benefit as it does not act as a process aimed at promoting survival through the detection of consecrated tissue damage [1].

Approximately 20% of the adult European population suffers from chronic pain, and among the top four causes of years lost due to disability, three (low back pain, musculoskeletal disorders, and neck pain) are conditions associated with chronic pain [1,2,3]. Chronic pain not only leads to disability, anxiety, depression, sleep disorders, and poor quality of life [4], but also imposes a significant economic burden on society, estimated at over €200 billion annually in Europe and $150 billion annually in the US [3].

Regarding its management, satisfactory results are not being achieved, as up to two-thirds of patients report dissatisfaction with treatment, and in many cases, the pain persists for years [3]. One current issue in chronic pain management is that healthcare professionals have traditionally focused on a biomedical approach to pain, with pharmacology as the primary strategy, without addressing potential non-pharmacological approaches such as physical exercise. However, the guidelines of the National Institute for Health and Care Excellence (NICE) establish that physical exercise should be a central treatment, [4] highlighting its analgesic effect, improvement of physical function, and mood in conditions involving non-oncological musculoskeletal chronic pain, such as osteoarthritis, fibromyalgia, idiopathic chronic low back pain, or idiopathic chronic neck pain, among others [5].

Measuring pain as a symptom is a challenge, as it predominantly relies on the use of subjective, self-reported scales to assess pain perception [6]. Due to this subjective nature of pain evaluation, the limited efficacy of treatment options, and the risks associated with opioid use, the need for objective data in the diagnostic and treatment processes for chronic pain becomes particularly relevant, including the use of biomarkers [7].

A biomarker is defined as an objective indicator that can be used to measure biological processes. According to the National Institutes of Health (NIH), “biomarkers could provide a means to objectify an inherently subjective measurement. In addition to identifying pain states and helping clinicians classify pain, biomarkers could guide treatment and prognosis and help predict treatment effectiveness and risks” [8].

Currently, a wide range of biomarkers for chronic pain is being investigated, ranging from the electrophysiology of peripheral nerves and the central nervous system, biochemical and “omics” analyses of blood, urine, saliva, cerebrospinal fluid, and other tissues, to structural and functional brain imaging [6]. Among these biomarkers are neurotransmitters such as β-endorphins, involved in the modulation of nociceptive circuits, acting on brainstem structures (the periaqueductal gray matter and the rostral ventromedial medulla) and the spinal cord [9]. Proinflammatory cytokines, such as IL-1β, IL-6, and TNFα, which can be objectively measured in the central nervous system and circulation, have been associated with central sensitization processes and the chronic pain experience [10].

Studies using modern neuroimaging techniques, such as functional magnetic resonance imaging (fMRI) and positron emission tomography (PET), have identified brain regions involved in sensory pain processing [11,12]. Conventional MRI is not a reliable biomarker of inflammation and, without clinical correlation, may misrepresent pain [13]. Furthermore, in recent years, interest in extracellular circulating vesicles and their role in various pain states has increased, with exosomes attracting the most attention, as their involvement in pain processes has been demonstrated in studies on various diseases such as osteoarthritis, rheumatoid arthritis, and complex regional pain syndrome [14].

Regarding the combination of physical exercise, non-oncological musculoskeletal chronic pain, and biomarkers, various studies have addressed the issue. However, it has been observed that physical exercise has been combined with other interventions such as diet or physiotherapy techniques, uncontrolled clinical trials have been evaluated, or chronic pain has been assessed as part of composite questionnaires or scales along with other variables, without providing isolated data for chronic pain [15,16]. Kawi et al. presented a more comprehensive approach to the issue addressed in this work [17]. However, they included studies based on different methodologies and not exclusively randomized controlled trials.

Therefore, the objective of this systematic review is to study the effect of physical exercise on non-oncological musculoskeletal chronic pain and its associated biomarkers based on randomized controlled trials.

## 2. Materials and Methods

This review was conducted following the PRISMA (Preferred Reporting Items for Systematic Reviews and Meta-Analyses) statement published in 2020 for systematic reviews [18] (see Supplementary Material, PRISMA 2020 and Abstract checklist). The execution of this study was approved by the Responsible Research Office (authorization code: TFG.GME.PZH.ICB.240926) of the Miguel Hernández University of Elche, and the study protocol was registered on the PROSPERO platform (CRD42025633310).

An electronic search was performed in the PubMed, Web of Science, and Scopus databases during January 2025. The keywords used were: “exercise,” “training,” “pain,” “musculoskeletal,” and “biomarkers,” combined in various ways using the Boolean operators “AND” and “OR.” The specific and complete search strategy for each database is provided in Supplementary Materials (Table S1).

**Figure 1:**
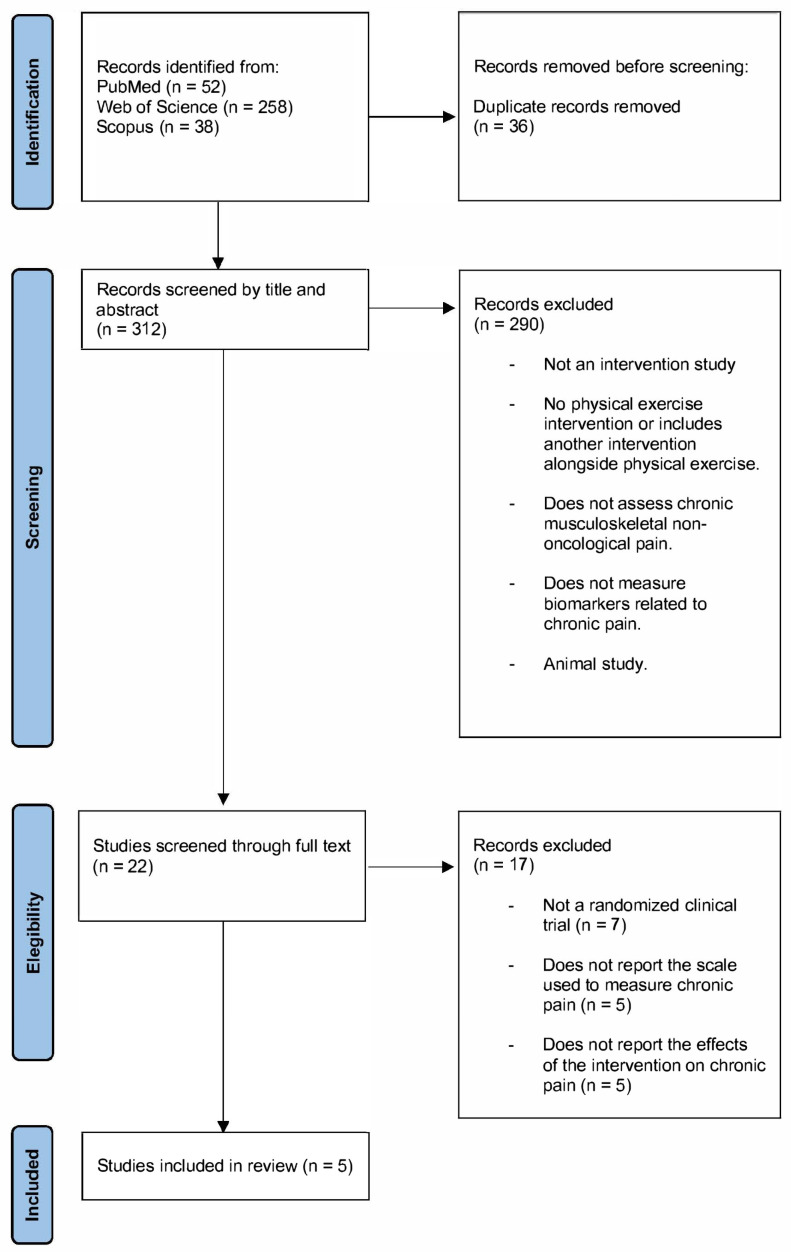
Flowchart of the literature search according PRISMA statement.

Once the search results were obtained, the strategy shown in Figure 1 was followed to identify the studies ultimately included in the review, with references managed using the EndNote x9 software.

### 2.1. Eligibility Criteria

The inclusion criteria were as follows: study methodology: randomized controlled trial; publication date: between 2004 and 2024; confirmed diagnosis: of a condition with non-oncological musculoskeletal chronic pain; intervention: involving physical exercise; measurement: of biomarkers associated with chronic pain.

The exclusion criteria were: presence of other pathologies or comorbidities; use of pharmacological interventions; measurement of biomarkers unrelated to chronic pain; and articles in a non-English language.

### 2.2. Study Selection and Data Extraction

**Figure 2:**
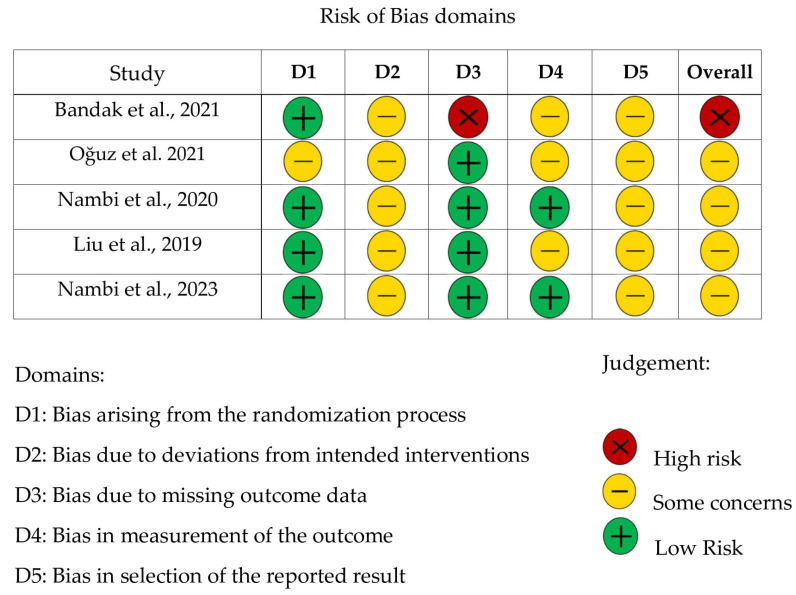
Risk of bias assessment for the randomized clinical trial studies included [19,20,21,22,23].

Once the eligibility criteria were defined, as shown in Figure 1, a total of 312 publications were screened by title and abstract by ICB, resulting in 22 publications selected for full-text review. Two authors, I.C.B. and T.Z., jointly assessed these publications, finalizing the selection of 5 studies to be included in this review. From the 5 selected publications, I.C.B. extracted the necessary data to determine the risk of bias in the studies, as shown in Figure 2.

### 2.3. Data Synthesis

Given the limited number of eligible studies (n = 5) and the substantial heterogeneity in study design, participant characteristics, exercise modalities, biomarkers assessed, and pain outcomes, a quantitative meta-analysis was not feasible. Instead, we adopted a narrative synthesis approach, following recommendations for systematic reviews where pooling of data is inappropriate. The results were structured by grouping findings according to biomarker categories (e.g., inflammatory markers, neurotrophic markers, cartilage metabolism markers, and imaging-based biomarkers) and their reported associations with pain outcomes. This approach allowed us to highlight patterns and inconsistencies across studies while maintaining methodological transparency.

## 3. Results

The initial literature search yielded a total of 348 results, of which 36 were duplicates and subsequently removed. A total of 312 publications were then screened by title and abstract, with 290 being excluded.

Finally, 22 publications were assessed in full text, with 17 being excluded, leaving 5 publications included in this review. The reasons for exclusion and the complete selection process are detailed in Figure 1.

### 3.1. Risk of Bias Assessment

The risk of bias of the included studies was assessed using the RoB 2 tool (Revised Cochrane risk-of-bias tool for randomized trials) proposed by Sterne et al. [24] (see Figure 2). This framework evaluates five domains (randomization, deviations from intended interventions, missing outcome data, outcome measurement, and selection of reported results), with judgments categorized as “low risk,” “some concerns,” or “high risk.” The overall risk of bias is determined by the highest level of concern across domains, with studies classified as low risk only if all domains are rated low, as some concerns if at least one domain raises concerns and none are high risk, and as high risk if any domain is rated high or if multiple domains raise concerns that substantially lower confidence in the results. A detailed interpretation of the risk of bias assessment is provided in Supplementary Materials (Table S2).

### 3.2. Characteristics of the Studies

The studies included were published between 2019 and 2023. The study conducted by Liu et al. [23] has the largest sample size (N = 108), while the study by Oğuz et al. [20] has the smallest sample size (N = 22). The age of participants ranges from 21.9 ± 1.3 years to 70 years.

Regarding gender, 3 of the studies, with a combined sample size of 190 participants, report 153 women (80.5%) and 37 men (19.5%), while 2 studies do not report the gender of participants.

The chronic pain-related conditions reported by these studies include primary knee osteoarthritis, post-traumatic knee osteoarthritis, and idiopathic chronic low back pain.

Regarding the physical exercise interventions, various modalities were found, such as functional exercises, Tai Chi, Baduanjin, stationary bike, muscle strengthening exercises, balance and stability exercises, stretching, virtual reality exercise, and sensorimotor exercise.

The duration of the interventions ranges from 4 weeks in Nambi et al. [21] to 12 weeks in Bandak et al. [19].

The chronic pain-related biomarkers identified in the studies can be classified as follows:

- Imaging-based biomarkers: magnetic resonance imaging with and without contrast, and functional brain magnetic resonance imaging.
- Inflammatory and immune response regulation biomarkers: IL-2, IL-4, IL-6, IL-10, IFN-γ, PD-1, TIM-3, CRP, TNF-α.
- Neurotrophic biomarkers: BDNF.
- Cartilage metabolism-related biomarkers: COMP, MMP-1, MMP-3.
- Bone morphogenetic proteins: BMP-2, BMP-4, BMP-6, BMP-7.

Finally, it is worth mentioning the instruments used to assess self-reported pain, including the KOOS (Knee Injury and Osteoarthritis Outcome Score) scale and the VAS (Visual Analog Scale).

A detailed analysis of the results found for the relationship between physical exercise, musculoskeletal chronic pain, and biomarkers in each of the reviewed studies can be found in Table 1.

Table 2 presents conclusions on the applicability of each examined biomarker, derived from the findings of the analyzed studies.

## 4. Discussion

The aim of this systematic review is to synthesize the available evidence from randomized controlled trials on the effect of physical exercise on non-oncological musculoskeletal chronic pain and its relationship with various biomarkers, and determine whether we can currently make use of measurements that help reduce the subjective component of pain.

The findings show that physical exercise, in various modalities, leads to a decrease in the perceived intensity of chronic pain [19,20,21,23]. However, not all biomarkers show changes consistent with the variation in pain [19,20,21]. Biomarkers related to inflammation [19,21,22] and functional and structural brain imaging biomarkers [23] seem to show a better correlation with pain reduction, with no relationship found between this reduction and biomarkers related to cartilage metabolism or bone morphogenetic proteins [20,21].

A key limitation of the available evidence is the marked heterogeneity across studies in terms of pain etiology, biomarkers assessed, and outcome measures. Osteoarthritis is now understood as a heterogeneous condition with distinct endotypes driven by inflammatory, metabolic, or post-traumatic mechanisms [25], which is reflected in the diverse biomarkers explored, including markers of cartilage and bone turnover, inflammatory mediators, and proteomic or metabolomic signatures [26]. Pain assessment methods also varied widely (e.g., WOMAC, KOOS, VAS), reducing comparability and consistency of findings [27]. Moreover, biomarker profiles differ according to etiology: cartilage and bone metabolism markers predominate in osteoarthritis, whereas inflammatory cytokines and neurodegenerative proteins are more relevant in spinal disorders, with further differences between disc herniation and spinal stenosis [28]. Such variability underscores the need for future trials to stratify populations by condition and to apply harmonized biomarker panels and standardized outcome measures in order to improve comparability and strengthen conclusions.

These findings align with some arguments suggesting that, in the search for biomarkers for chronic pain, the properties of peripheral tissue may be an inadequate source of information, as they provide insight into a nociceptive abnormality but are unable to capture the subjective experience of pain, which varies over time and context, being continuously modulated by recent experiences. Thus, pain is a subjective and conscious perception distinct from nociception [29]. Ultimately, currently available biomarkers associate pain with structural and molecular alterations rather than exploring, as some authors have suggested, the neurological processes involved in pain processing [30]. Pain biomarkers predominantly focus on peripheral structural and molecular changes, such as cartilage and bone degradation in osteoarthritis, yet they often fail to capture the complexity of the neurobiological mechanisms underlying the pain experience. Thudium et al. reviewed protein biomarkers associated with osteoarthritis pain, clearly highlighting this discrepancy [30]. From a comparative approach between genders, it can be observed that women are more represented in the studies. The prevalence of musculoskeletal disorders differs between men and women, and a response to this fact is currently being sought. Explanations based on gender differences, such as the influence of reduced estrogen after menopause, the unequal functioning of the immune system, and differences in pain perception [31,32], have been proposed, as well as gender-based differences, since sociocultural influences, such as gender roles, expectations, and social learning, have been shown to contribute to differences in pain experience [33].

A strength of this review is the inclusion of studies based on the methodology of randomized controlled trials, considered the gold standard for establishing causal relationships, and to date, this is the only study that has evaluated this research question from this perspective.

The results obtained suggest various practical implications. On one hand, physical exercise is an effective tool for reducing the perception of chronic pain, and it is generally a low-cost and accessible strategy for patients, so it should be integrated into multimodal pain management plans. On the other hand, physical exercise may be a useful tool for evaluating biomarkers related to chronic pain, as it acts as a stimulus that induces physiological adaptations in various body systems, such as the immune system or central nervous system, exerting its effect on pain modulation, establishing it as a natural experimental model.

Future research could consider studies with longer intervention durations, as adaptations and the reflection of these through biomarkers may evolve differently over time. Also, new studies could focus on the interaction between physical exercise and the changes experienced at the level of neurological processes involved in central pain modulation. Finally, due to the wide variety of types of physical exercise and their programming in terms of frequency, intensity, and volume, greater emphasis should be placed on determining which exercise modalities are most involved in pain perception and have the greatest impact on known biomarkers associated with chronic pain. Indeed, such heterogeneity makes it difficult to attribute biomarker or pain-related changes to a specific exercise modality, emphasizing the importance of developing standardized protocols and conducting direct head-to-head comparisons in future research. A recent systematic review similarly noted that variability in exercise types, biomarkers assessed, and outcome measures limits the capacity to draw uniform conclusions regarding the effects of exercise interventions, thereby reinforcing the need for more standardized and rigorously designed studies [34].

### Limitations

This review presents several limitations that warrant consideration. First, the included randomized trials were heterogeneous with respect to pain etiology (e.g., primary and post-traumatic knee osteoarthritis, idiopathic chronic low back pain), the biomarkers assessed (inflammatory mediators, cartilage turnover, neurotrophic factors, and imaging-based measures), and the tools used for pain evaluation. Such variability hampers comparability across studies and prevents the formulation of uniform conclusions. Second, the exercise interventions differed substantially, ranging from Tai Chi, Baduanjin, and cycling to functional strengthening, stretching, virtual reality, and sensorimotor training. This diversity complicates attribution of biomarker or pain-related changes to a specific modality, underscoring the need for standardized protocols or direct head-to-head comparisons in future research. Third, although imaging biomarkers such as MRI and fMRI were included, these techniques cannot be regarded as direct indicators of inflammation or pain localization. As shown in prior literature and reaffirmed here, MRI findings often fail to correspond with the clinical pain experience, requiring careful interpretation alongside clinical and functional assessments. Fourth, biomarkers alone do not capture the full complexity of patient status; only integration with classical inflammatory signs and functional testing can provide a comprehensive evaluation. Finally, most trials were of short duration (≤12 weeks), potentially insufficient to detect long-term adaptations, and some eligible studies may have been missed due to database restrictions and the specific selection of keywords and Boolean operators.

## 5. Conclusions

According to this systematic review of randomized controlled trials, physical exercise could be an effective intervention for reducing pain perception in patients with non-oncological musculoskeletal chronic pain. While not all associated biomarkers show consistent changes, those related to inflammation and functional brain activity appear to be more correlated with pain reduction. These findings highlight the importance of integrating exercise into pain management plans and suggest that it may be a useful experimental model for exploring complex biological mechanisms associated with chronic pain.

The results underscore the complexity of the chronic pain phenomenon and emphasize the importance of designing future studies with robust methodologies and multidimensional approaches to better understand the underlying mechanisms.

## Figures and Tables

**Table 1:** Analysis of study characteristics and outcomes.

Authors (Year)	Sample	Objective	Intervention	Biomarkers	Studied Variable	Results
Bandak et al., (2021) [19]	N = 60; intervention group = 31 (27 women, 4 men; age = 65.9); control group = 29 (21 women, 8 men; age = 61.3).	To determine the effect of physical exercise on inflammatory activity and the relationship between biomarkers of structural changes and reported pain in individuals with knee osteoarthritis.	- Intervention group: Functional exercise sessions (duration not reported), 3 times a week for 12 weeks. - Control group: No intervention.	Knee MRI with and without contrast, IL-6, IL-10	Pain reported using the KOOS pain subscale	Statistically significant reduction in perceived pain in the intervention group compared to the control group (*p* = 0.0075). No statistically significant differences between groups in MRI changes with contrast (*p* = 0.052) and without contrast (*p* = 0.122), IL-6 (*p* = 0.672), and IL-10 (*p* = 0.871).
Liu et al., (2019) [23]	N = 108; Tai Chi group = 28 (22 women, 6 men; age = 40–70); Baduanjin group = 29 (24 women, 5 men; age = 40–68); static bicycle group = 27 (23 women, 4 men; age = 40–70); control group = 24 (14 women, 10 men; age = 40–70)	To determine the effect of different physical exercise modalities on pain, changes in brain regions associated with the opioid and reward/motivation system, and immune and inflammatory biomarkers in patients with knee osteoarthritis.	- Tai Chi group: 1 h sessions, 5 times a week for 12 weeks. - Baduanjin group: 1 h sessions, 5 times a week for 12 weeks. - Static bicycle group: 1 h sessions, 5 times a week for 12 weeks. - Control group: No intervention.	Functional brain MRI, BDNF, IFN-γ, PD-1, TIM-3	Pain reported using the KOOS pain subscale	Compared to the control group: - Statistically significant reduction in perceived pain (Tai Chi, *p* < 0.01; Baduanjin, *p* < 0.01; static bicycle, *p* = 0.05). - Significant reductions in PD-1 concentrations in the Baduanjin (*p* = 0.05) and static bicycle groups (*p* < 0.01), and in IFN-γ levels across all intervention groups (*p* < 0.01). - Significant reduction in resting-state functional connectivity between the right periaqueductal gray area and medial orbitofrontal cortex (no *p*-value reported). - Significant increase in gray matter volume in the medial orbitofrontal cortex in all intervention groups (Tai Chi, *p* = 0.003; Baduanjin, *p* = 0.048; static bicycle, *p* = 0.03).
Oğuz et al., (2021) [20]	N = 22; physical exercise group = 11 (11 women; age = 51 ± 3.69); physical exercise + kinesio-taping group = 11 (11 women; age = 48.18 ± 7.56)	To investigate the effects of physical exercise alone or in combination with kinesio-taping on pain, functionality, and biomarkers related to cartilage metabolism in patients with knee osteoarthritis.	- Physical exercise group: Strengthening, balance/stability, and stretching exercises for 60 min, 3 times a week for 6 weeks. - Physical exercise + kinesio-taping group: same exercise protocol.	COMP, MMP-1, MMP-3	Pain reported using the VAS scale	Statistically significant reduction in perceived pain in both groups (*p* < 0.05), with no statistically significant differences between groups (*p* > 0.05). No statistically significant changes in COMP, MMP-1, or MMP-3 concentrations in the physical exercise group, with no significant differences between groups (*p* > 0.05).
Nambi et al., (2020) [21]	N = 60; virtual reality training group = 20, age = 22.8 ± 1.3; sensori-motor training group = 20, age = 22.6 ± 1.4; control group = 20, age = 21.9 ± 1.3. Sex not reported.	To compare the effects of virtual reality training and sensori-motor training on bone morphogenetic proteins and inflammatory biomarkers in individuals with post-traumatic knee osteoarthritis.	- Virtual reality training group: 2 sessions of 20 min, 5 days a week for 4 weeks. - Sensori-motor training group: 3 exercises, 5 repetitions per set, 3 sets with 3 min of rest between sets, 5 days a week for 4 weeks. - Control group: conventional knee exercises, 10–15 repetitions per set, 3 sets with 1 min of rest between sets, 5 days a week for 4 weeks.	BMP-2; BMP-4; BMP-6; BMP-7; CRP; TNF-α, IL-2; IL-4; IL-6	Pain reported using the VAS scale	Significant reduction in pain intensity in the virtual reality training group compared to the control group (*p* < 0.001). No statistically significant variations between groups in bone morphogenetic protein concentrations BMP-2 (*p* = 0.946), BMP-4 (*p* = 0.967), BMP-6 (*p* = 0.930), and BMP-7 (*p* = 0.924). Significant reduction in inflammatory biomarkers such as CRP (*p* = 0.001), TNF-α (*p* = 0.001), IL-2 (*p* = 0.015), IL-4 (*p* = 0.001), and IL-6 (*p* = 0.001) in the virtual reality training group compared to the other groups.
Nambi et al., (2023) [22]	N = 60; virtual reality training group = 20, age = 23.2 ± 1.6; isokinetic training group = 20, age 22.9 ± 1.7; control group = 20, age 22.8 ± 1.8. Sex not reported.	To investigate the effects of physical exercise on inflammatory biomarkers in individuals with idiopathic chronic low back pain.	For all three groups, sessions of their specific exercises lasted 30 min, 5 days a week for 4 weeks.	CRP; TNF-α, IL-2; IL-4; IL-6	Pain reported using the VAS scale	Significant reduction in pain intensity in the virtual reality training group compared to the other groups (*p* = 0.001). Significant reduction in inflammatory biomarker concentrations such as CRP (*p* = 0.001), TNF-α (*p* = 0.001), IL-2 (*p* = 0.001), IL-4 (*p* = 0.001), and IL-6 (*p* = 0.001) in the virtual reality training group compared to the other groups.

Abbreviations: BDNF = brain-derived neurotrophic factor; BMP = bone morphogenic protein; COMP = cartilage oligomeric matrix protein; CRP = C-reactive protein; IL = interleukin; INF-γ = interferon γ; KOOS = Knee Injury and Osteoarthritis Outcome Score; MMP = matrix metalloproteinase; MRI = magnetic resonance imaging; PD-1 = programmed death 1; TIM-3 = T-cell Ig- and mucin-domain-containing molecule-3; TNF-α = tumor necrosis factor α; VAS = visual analogue scale.

**Table 2:** Conclusions derived from the analysis of biomarkers.

Authors (Year)	Biomarker	Conclusion
Bandak et al., (2021) [19]	Knee MRI with and without contrast IL-6, IL-10	Report the presence of synovitis. No association is identified between the reduction in pain perception and inflammatory activity at the synovial level. This suggests that changes in pain perception may be due to other mechanisms, such as the systemic anti-inflammatory effect and/or improved psychological well-being. Report systemic pro-inflammatory activity. No association is identified between the reduction in pain perception and variations in the concentrations of these biomarkers. The observed pain reduction may be attributed to other mechanisms, such as improved muscle strength, joint range of motion, or enhanced proprioception.
Liu et al., (2019) [23]	Functional brain MRI BDNF IFN-γ PD-1 TIM-3	An association is identified between the reduction in pain perception and functional changes recorded in the brain in areas related to descending pain modulation as well as reward/motivation systems. Physical exercise may modulate brain areas involved in opioid and dopaminergic neurotransmission systems to alleviate pain. Its plasma concentrations have been linked to self-reported pain. No association is identified between the reduction in pain perception and the plasma concentrations of this biomarker, as no changes in its concentration are observed. It has been associated with spinal microglia activation, triggering pain, and has also been linked to systemic inflammatory activity. An association is identified between the reduction in pain perception and the decrease in the concentrations of this inflammatory activity biomarker, reflecting the relationship between perceived pain and systemic inflammatory activity. It plays a role in regulating the immune response by decreasing its activity. An association is identified between the reduction in pain perception and the increase in the concentrations of this immune response biomarker, reflecting the relationship between perceived pain and the level of immune system activation. It is involved in the regulation of the immune system and the inflammatory response. No association is identified between the reduction in pain perception and the plasma concentrations of this biomarker, as no changes in its concentration are observed.
Oğuz et al., (2021) [20]	COMP MMP-1, MMP-3	Report the degradation of articular cartilage. No association is identified between the reduction in pain perception and this biomarker, which does not change in concentration, suggesting that cartilage metabolism may not be affected. Report the degradation of the extracellular matrix in synovial tissue and articular cartilage. No association is identified between the reduction in pain perception and variations in the concentrations of these biomarkers. While MMP-1 remains unchanged after the intervention period, MMP-3 decreases. Physical exercise improves pain perception independently of variations in these biomarkers of articular cartilage.
Nambi et al., (2020) [21]	BMP-2, BMP-4, BMP-6, BMP-7 CRP, TNF-α, IL-2, IL-4, IL-6	Exert an anabolic effect on chondrocytes by activating the synthesis of cartilage matrix components, regulating and promoting the synthesis of proteoglycans and collagen, and playing a positive role in inflammatory cytokines. No association is identified between the reduction in pain perception and the concentration of these biomarkers. Report on the systemic inflammatory response. An association is identified between the reduction in pain perception and the decrease in the concentrations of these biomarkers, reflecting the relationship between perceived pain and systemic inflammatory activity.
Nambi et al., (2023) [22]	CRP, TNF-α, IL-2, IL-4, IL-6	Report on the systemic inflammatory response. An association is identified between the reduction in pain perception and the decrease in the concentrations of these biomarkers, reflecting the relationship between perceived pain and systemic inflammatory activity.

Abbreviations: BDNF = brain-derived neurotrophic factor; BMP = bone morphogenic protein; COMP = cartilage oligomeric matrix protein; CRP = C-reactive protein; IL = interleukin; INF-γ = interferon γ; MMP = matrix metalloproteinase; MRI = magnetic resonance imaging; PD-1 = programmed death 1; TIM-3 = T-cell Ig- and mucin-domain-containing molecule-3; TNF-α = tumor necrosis factor α.

## Data Availability

The data that support the findings of this study are available from the corresponding author upon reasonable request.

## Supplementary Materials

The following supporting information can be downloaded at https://www.mdpi.com/article/10.3390/life15091413/s1, Table S1. Research strategy; Table S2. Summary of risk of bias assessment for five samples included studies using Cochrane risk of bias assessments (RoB 2).

## Abbreviations

The following abbreviations are used in this manuscript:

BDNF	Brain-Derived Neurotrophic Factor
BMP	Bone Morphogenic Protein
COMP	Cartilage Oligomeric Matrix Protein
CRP	C-reactive Protein
IASP	International Association for the Study of Pain
IL	Interleukin
INF-γ	Interferon γ
KOOS	Knee Injury and Osteoarthritis Outcome Score
MMP	Matrix Metalloproteinase
MRI	Magnetic Resonance Imaging
NICE	National Institute for Health and Care Excellence
NIH	National Institutes of Health
PET	Positron Emission Tomography
PD-1	Programmed Death 1
TIM-3	T-Cell Ig- And Mucin-Domain-Containing Molecule-3
TNF-α	Tumor Necrosis Factor A
VAS	Visual Analogue Scale

## References

[1] Cohen S.P., Vase L., Hooten W.M. (2021). Chronic Pain: An Update on Burden, Best Practices, and New Advances. Lancet.

[2] Crofford L.J. (2015). Chronic Pain: Where the Body Meets the Brain. Trans. Am. Clin. Climatol. Assoc..

[3] Van Hecke O., Torrance N., Smith B.H. (2013). Chronic Pain Epidemiology and Its Clinical Relevance. Br. J. Anaesth..

[4] Geneen L., Moore R., Clarke C., Martin D., Colvin L., Smith B. (2017). Physical Activity and Exercise for Chronic Pain in Adults: An Overview of Cochrane Reviews. Cochrane Database Syst. Rev..

[5] Rice D., Nijs J., Kosek E., Wideman T., Hasenbring M., Koltyn K., Graven-Nielsen T., Polli A. (2019). Exercise-Induced Hypoalgesia in Pain-Free and Chronic Pain Populations: State of the Art and Future Directions. J. Pain.

[6] Eldabe S., Obara I., Panwar C., Brookes M., Gulve A., Klukinov M., Hegarty D., Johnson M.I. (2022). Biomarkers for Chronic Pain: Significance and Summary of Recent Advances. Pain Res. Manag..

[7] Gunn J., Hill M.M., Cotten B.M., Mittal D., Aouizerat B.E., Finley P.J. (2020). An Analysis of Biomarkers in Patients with Chronic Pain: An Observational Study. Pain Physician.

[8] Levitt J., Saab C.Y. (2019). What Does a Pain ‘Biomarker’ Mean and Can a Machine Be Taught to Measure Pain?. Neurosci. Lett..

[9] De Assis E.B., De Carvalho C.D., Martins C., Diniz L.R.L., de Oliveira L.R.C., Silva L.R., Macêdo M.C.S., Leite H.R. (2021). Beta-Endorphin as a Biomarker in the Treatment of Chronic Pain with Non-Invasive Brain Stimulation: A Systematic Scoping Review. J. Pain Res..

[10] Pinto E.M., Neves J.R., Laranjeira M., Almeida L., Silva R., Gonçalves J., Fernandes R., Sousa R. (2023). The Importance of Inflammatory Biomarkers in Non-Specific Acute and Chronic Low Back Pain: A Systematic Review. Eur. Spine J..

[11] Kashanian A., Tsolaki E., Caruso J., Kasper E.M. (2022). Imaging as a Pain Biomarker. Neurosurg. Clin. N. Am..

[12] Rockholt M.M., Kenefati G., Doan L.V., Llinas R.H., Agrawal A., Zhou Y. (2023). In Search of a Composite Biomarker for Chronic Pain by Way of EEG and Machine Learning: Where Do We Currently Stand?. Front. Neurosci..

[13] Vagaska E., Litavcova A., Srotova I., Kralovicova L., Kocmalova M., Madarasova-Geckova A., van Dijk J.P., Reijneveld S.A., Rosenberger J. (2019). Do Lumbar Magnetic Resonance Imaging Changes Predict Neuropathic Pain in Patients with Chronic Non-Specific Low Back Pain?. Medicine.

[14] D’Agnelli S., Gerra M.C., Bignami E., Arendt-Nielsen L. (2020). Exosomes as a New Pain Biomarker Opportunity. Mol. Pain.

[15] Aguiar G.C., do Nascimento M.R., de Miranda A.S., Rocha N.P., Teixeira A.L., Scalzo P.L. (2015). Effects of an Exercise Therapy Protocol on Inflammatory Markers, Perception of Pain, and Physical Performance in Individuals with Knee Osteoarthritis. Rheumatol. Int..

[16] Uzunel E., Kronhed A.C.G., Alin C.K., Ahlgren C., Salminen H. (2023). The Effect of Group Training or Spinal Orthosis on Quality of Life and Potential Plasma Markers of Pain in Older Women with Osteoporosis: A Randomized Controlled Trial. Arch. Rehabil. Res. Clin. Transl..

[17] Kawi J., Lukkahatai N., Inouye J., Hinds P., Saligan L. (2016). Effects of Exercise on Select Biomarkers and Associated Outcomes in Chronic Pain Conditions: Systematic Review. Biol. Res. Nurs..

[18] Page M.J., McKenzie J.E., Bossuyt P.M., Boutron I., Hoffmann T.C., Mulrow C.D., Shamseer L., Tetzlaff J.M., Akl E.A., Brennan S.E. (2021). The PRISMA 2020 Statement: An Updated Guideline for Reporting Systematic Reviews. BMJ.

[19] Bandak E., Boesen M., Bliddal H., Brix M., Henriksen M. (2021). The Effect of Exercise Therapy on Inflammatory Activity Assessed by MRI in Knee Osteoarthritis: Secondary Outcomes from a Randomized Controlled Trial. Knee.

[20] Oğuz R., Belviranlı M., Okudan N. (2021). Effects of Exercise Training Alone and in Combination with Kinesio Taping on Pain, Functionality, and Biomarkers Related to Cartilage Metabolism in Knee Osteoarthritis. Cartilage.

[21] Nambi G., Abdelbasset W.K., Elsayed S.H., Elnegamy T.E., Alqahtani B.A., Ibrahim A.A., Verma A., Venugopal A., Aldhafian O.R., Kakaraparthi V.N. (2020). Comparative Effects of Virtual Reality Training and Sensory Motor Training on Bone Morphogenic Proteins and Inflammatory Biomarkers in Post-Traumatic Osteoarthritis. Sci. Rep..

[22] Nambi G., Alghadier M., Kashoo F.Z., Alqahtani B.A., Aldhafian O.R., Alnahdi A.H., Alsubaie S.F., Alghamdi M.S., Abdelbasset W.K. (2023). Effects of Virtual Reality Exercises versus Isokinetic Exercises in Comparison with Conventional Exercises on the Imaging Findings and Inflammatory Biomarker Changes in Soccer Players with Non-Specific Low Back Pain: A Randomized Controlled Trial. Int. J. Environ. Res. Public Health.

[23] Liu J., Chen L., Chen X., Hu K., Tu Y., Lin M., Kong J. (2019). Modulatory Effects of Different Exercise Modalities on the Functional Connectivity of the Periaqueductal Grey and Ventral Tegmental Area in Patients with Knee Osteoarthritis: A Randomised Multimodal Magnetic Resonance Imaging Study. Br. J. Anaesth..

[24] Sterne J.A.C., Savović J., Page M.J., Elbers R.G., Blencowe N.S., Boutron I., Cates C.J., Cheng H.-Y., Corbett M.S., Eldridge S.M. (2019). RoB 2: A Revised Tool for Assessing Risk of Bias in Randomised Trials. BMJ.

[25] Robinson W.H., Lepus C.M., Wang Q., Raghu H., Mao R., Lindstrom T.M., Sokolove J. (2016). Low-Grade Inflammation as a Key Mediator of the Pathogenesis of Osteoarthritis. Nat. Rev. Rheumatol..

[26] Mobasheri A., Bay-Jensen A.C., van Spil W.E., Larkin J., Levesque M.C. (2017). Osteoarthritis Year in Review 2016: Biomarkers (Biochemical Markers). Osteoarthr. Cartil..

[27] McAlindon T.E., Driban J.B., Henrotin Y., Hunter D.J., Jiang G.L., Skou S.T., Wang S.X., Schnitzer T.J. (2015). OARSI Clinical Trials Recommendations: Design, Conduct, and Reporting of Clinical Trials for Knee Osteoarthritis. Osteoarthr. Cartil..

[28] Khan A.N., Jacobsen H.E., Khan J., Filippi C.G., Levine M., Lehman R.A., Riew K.D., Lenke L.G., Chahine N.O. (2017). Inflammatory Biomarkers of Low Back Pain and Disc Degeneration: A Review. Ann. N. Y. Acad. Sci..

[29] Reckziegel D., Vachon-Presseau E., Petre B., Schnitzer T.J., Baliki M.N., Apkarian A.V. (2019). Deconstructing Biomarkers for Chronic Pain: Context- and Hypothesis-Dependent Biomarker Types in Relation to Chronic Pain. Pain.

[30] Thudium C.S., Löfvall H., Karsdal M.A., Bay-Jensen A.C., Bihlet A.R. (2019). Protein Biomarkers Associated with Pain Mechanisms in Osteoarthritis. J. Proteom..

[31] Overstreet D.S., Strath L.J., Jordan M., Quinn T.L., LaVigne T., DeVon H.A., Goodin B.R. (2023). A Brief Overview: Sex Differences in Prevalent Chronic Musculoskeletal Conditions. Int. J. Environ. Res. Public Health.

[32] Wolf J.M., Cannada L., Van Heest A.E., O’Connor M.I., Ladd A., Goldfarb C.A., Gelberman R.H. (2015). Male and Female Differences in Musculoskeletal Disease. J. Am. Acad. Orthop. Surg..

[33] Pedulla R., Glugosh J., Jeyaseelan N., Nicolson P.J.A., Sharma S., Hall A., Costa M.L. (2024). Associations of Gender Role and Pain in Musculoskeletal Disorders: A Mixed-Methods Systematic Review. J. Pain.

[34] Alonso-Sal A., Alonso-Perez J.L., Sosa-Reina M.D., Silva A., Cuesta-Vargas A.I. (2024). Effectiveness of Physical Activity in the Management of Nonspecific Low Back Pain: A Systematic Review. Medicina.

