# Supplementary material for: The Effect of Physical Exercise on Non-Oncological Musculoskeletal Chronic Pain and Its Associated Biomarkers: Systematic Review on Randomized Controlled Trials
Source: Life (Basel). 2025 Sep 8;15(9):1413. doi: 10.3390/life15091413 (PMC12471879; doi:10.3390/life15091413)
Supplement: Supplementary file 1 [file life-15-01413-s001.zip › life-3831050-supplementary.pdf]

The effect of physical exercise on non-oncological musculoskeletal chronic pain and its associated biomarkers: systematic review on randomized controlled trials.

Supplementary materials

Table S1. Search strategy.

**Database**	**Keywords**	**Search combination**	**Results**
PUBMED	"exercise"; "training"; "pain"; "musculoskeletal"; "biomarkers"	(((exercise[Title/Abstract]) OR (training[Title/Abstract])) AND (pain[Title/Abstract])) AND (biomarkers[Title/Abstract])<br><br>Filters applied: randomized clinical trial; English; 2004-2024	52
WEB OF SCIENCE		exercise (Topic) AND pain (Topic) AND biomarkers (Topic)<br><br>Filters applied: article; English; 2004-2024	258
SCOPUS		(TITLE-ABS-KEY (exercise) AND TITLE-ABS-KEY (pain) AND TITLE-ABS-KEY (biomarkers) AND TITLE-ABS-KEY (musculoskeletal)<br><br>Filters applied: article; English; 2004-2024	38

Table S2. Summary of risk of bias assessment for five samples included studies using Cochrane risk of bias assessments (RoB 2).

Study	**Domains**	**Comments**	**Judgment**

Bandak et al., 2021	1. Bias arising from the randomization process	Random allocation (1:1) using computer-generated sequence; stratification by sex; concealment mentioned but not detailed ("concealed from the researchers enrolling and assessing participants").	Low risk
	2. Bias due to deviations from intended interventions	Participants were aware of group allocation (open-label design). Exercise group received supervised, individualized program; control group received no attention. Lack of blinding may influence adherence and self-reported outcomes.	Some concerns
	3. Bias due to missing outcome data	60 participants randomized; only 33 (ET=16, CG=17) included in per-protocol MRI analysis. No ITT analysis performed; exclusions due to adherence/non-valid MRI reduce representativeness.	High risk
	4. Bias in measurement of the outcome	MRI assessments performed by blinded readers. KOOS outcomes validated but self-reported, with participants aware of allocation, raising potential bias.	Some concerns
	5. Bias in selection of the reported result	Trial registered (NCT01545258). MRI outcomes were secondary. Multiple exploratory MRI/biomarker analyses tested in small sample without adjustment for multiplicity; selective emphasis possible.	Some concerns
	Overall risk of bias	Main limitation: high attrition and per-protocol analysis only, combined with lack of blinding and exploratory multiple outcomes.	High risk
Liu et al., 2019	1. Bias arising from the randomization process	Participants (n=140) were randomly assigned to Tai Chi, Baduanjin, cycling, or health education groups. Randomization described and balanced baseline characteristics (no significant differences in age, sex, KOOS sub-scores, or biomarkers at baseline).	Low risk
	2. Bias due to deviations from intended interventions	The trial was not blinded: participants and instructors knew group allocation. Exercise groups had supervised sessions, while the control group only received health education once a week. This may have introduced performance bias.	Some concerns
	3. Bias due to missing outcome data	108 of 140 participants (77%) completed the trial. Dropouts were reported, and no significant differences were found between completers and non-completers in baseline characteristics. Attrition balanced across groups.	Low risk
	4. Bias in measurement of the outcome	Clinical outcomes (KOOS pain and other subscales) were self-reported, with participants aware of group allocation. MRI and biomarker analyses were objective, but fMRI preprocessing steps could be sensitive to analytical choices.	Some concerns
	5. Bias in selection of the reported result	The trial was registered (ChiCTR-IOR-16009308), and outcomes (pain, MRI, biomarkers) were pre-specified. However, multiple MRI and biomarker outcomes were analyzed without adjustment for multiplicity, raising potential selective emphasis.	Some concerns

	Overall risk of bias	Although randomization and reporting were robust, lack of blinding of participants and reliance on multiple exploratory outcomes without multiplicity correction reduce confidence in the findings.	Some concerns
Oğuz et al., 2021	1. Bias arising from the randomization process	Randomization was performed using the sealed envelope method with equal group assignment (n=11 each). While randomization was described, allocation concealment is less robust with sealed envelopes, and no stratification was reported.	Some concerns
	2. Bias due to deviations from intended interventions	Participants and physiotherapists were aware of group allocation (exercise vs. exercise + kinesio taping). No blinding was applied, which could have influenced adherence or performance. Interventions were supervised, reducing risk of non-compliance.	Some concerns
	3. Bias due to missing outcome data	All 22 participants completed the 6-week intervention and post-tests, with no reported dropouts.	Low risk
	4. Bias in measurement of the outcome	Pain and function were assessed with self-reported VAS and WOMAC, without blinding of participants. Biomarker analysis (COMP, MMP-1, MMP-3) was objective and performed using ELISA kits, reducing risk for laboratory outcomes.	Some concerns
	5. Bias in selection of the reported result	The study focused on COMP, MMP-1, and MMP-3, which were clearly pre-specified in the objectives. However, trial registration was not reported, and the absence of an a priori analysis plan raises the possibility of selective reporting.	Some concerns
	Overall risk of bias	The study had strengths, including complete outcome data and supervised interventions. However, methodological limitations, open-label design, limited randomization concealment, and lack of trial registration, lower confidence in the robustness of results.	Some concerns
Nambi et al., 2020	1. Bias arising from the randomization process	Participants (n=60) were randomized into three groups (VRT, SMT, control) using a computer-generated random table, with allocation via sealed envelopes. Baseline demographics and clinical characteristics were comparable across groups.	Low risk
	2. Bias due to deviations from intended interventions	Participants were informed of group assignment, and physiotherapists delivering interventions were not blinded. However, interventions were supervised and standardized. Differences in intervention type (technology-based VRT vs. SMT vs. conventional training) may have influenced expectations.	Some concerns
	3. Bias due to missing outcome data	Dropout rates were low (2 participants in VRT, 2 in SMT, 1 in control). An intention-to-treat (ITT) analysis was reported, minimizing bias from attrition.	Low risk

	4. Bias in measurement of the outcome	Outcome assessors were blinded to group allocation. Participants were asked not to share intervention details with the evaluating therapist. Pain (VAS) and disability (WOMAC) are validated self-reports, while biomarkers were measured via ELISA, ensuring objectivity.	Low risk
	5. Bias in selection of the reported result	Outcomes (pain, WOMAC, BMP, inflammatory biomarkers) were pre-specified and reported as described. However, trial registration or a pre-published protocol was not reported, raising the possibility of selective emphasis on significant findings.	Some concerns
	Overall risk of bias	The study benefited from proper randomization, ITT analysis, and blinded outcome assessors. However, absence of participant/treating therapist blinding and lack of pre-registered protocol lead to an overall judgment of "some concerns."	Some concerns
Nambi et al., 2023	1. Bias arising from the randomization process	Participants (n=60) were randomly assigned (block randomization) into three groups: virtual reality exercise (VRE), isokinetic exercise (IKE), and conventional exercise (n=20 each). Allocation was performed by sealed envelopes handled by a blinded therapist. Baseline characteristics were comparable across groups.	Low risk
	2. Bias due to deviations from intended interventions	The treating physiotherapist and participants could not be blinded due to the nature of the interventions. Although supervision and standardized protocols were applied, awareness of group allocation could influence adherence and reporting.	Some concerns
	3. Bias due to missing outcome data	Only two participants dropped out (one each in the VRE and IKE groups), leaving 58/60 participants for analysis. Reasons for dropout were unrelated to intervention effects, and attrition was balanced across groups.	Low risk
	4. Bias in measurement of the outcome	The outcome assessor was blinded to group allocation, and participants were instructed not to reveal intervention details. Pain (VAS) is validated but self-reported, while imaging outcomes (MRI and ultrasound) and biomarker measures (ELISA) were objective.	Low risk
	5. Bias in selection of the reported result	The study was retrospectively registered (NCT05253599, February 2022), after data collection began, which increases risk of selective reporting. However, all major outcomes (pain, muscle CSA/thickness, inflammatory biomarkers) were reported as described in the Methods.	Some concerns
	Overall risk of bias	The trial had strong design features (randomization, blinded outcome assessment, low attrition), but retrospective registration and lack of participant blinding reduce confidence, resulting in an overall rating of "some concerns."	Some concerns
